# Evolution and expansion of the One Health approach to promote sustainable and resilient health and well-being: A call to action

**DOI:** 10.3389/fpubh.2022.1056459

**Published:** 2023-01-13

**Authors:** Elizabeth L. Mumford, Deniss J. Martinez, Karli Tyance-Hassell, Alasdair Cook, Gail R. Hansen, Ronald Labonté, Jonna A. K. Mazet, Elaine C. Mumford, David M. Rizzo, Eri Togami, Arioene Vreedzaam, John Parrish-Sprowl

**Affiliations:** ^1^School of Veterinary Medicine, University of Surrey, Guildford, United Kingdom; ^2^Graduate Group in Ecology, University of California, Davis, Davis, CA, United States; ^3^(Anishinaabe) Office of Research and Community Engagement, Alaska Pacific University, Anchorage, AK, United States; ^4^Department of Veterinary Epidemiology and Public Health, University of Surrey, Guildford, United Kingdom; ^5^Hansen Consulting, LLC, Washington, DC, United States; ^6^School of Epidemiology and Public Health, University of Ottawa, Ottawa, ON, Canada; ^7^Grand Challenges, University of California, Davis, Davis, CA, United States; ^8^Independent Researcher, Uppsala, Sweden; ^9^Department of Plant Pathology, University of California, Davis, Davis, CA, United States; ^10^Department of Environmental Health and Engineering, Bloomberg School of Public Health, Johns Hopkins University, Baltimore, MD, United States; ^11^Anton de Kom University of Suriname, Paramaribo, Suriname; ^12^Department of Communication Studies, Indiana University, Indianapolis, IN, United States

**Keywords:** One Health, traditional indigenous knowledge, sustainability, complexity, systems, worldview, community, ecology

## Abstract

One Health is a transdisciplinary approach used to address complex concerns related to human, animal, plant, and ecosystem health. One Health frameworks and operational tools are available to support countries and communities, particularly for the prevention and control of zoonotic diseases and antimicrobial resistance and the protection of food safety. However, One Health has yet to be implemented in a manner that fully considers the complexities and interconnectedness of the diverse influences that have impacts at a larger system level. This lack of consideration can undermine the sustainability of any positive outcomes. To ensure the One Health approach can function effectively within the new global context of converging and escalating health, social, economic, and ecological crises, it must evolve and expand in three overlapping dimensions: (1) Scope: the partners, knowledge, and knowledge systems included, (2) Approach: the techniques, methodologies, and scholarship considered, and (3) Worldview inclusivity: the interweaving of other worldviews together with the mainstream scientific worldview that currently predominates. Diverse partners and knowledge from outside the mainstream health and scientific sectors, including Indigenous peoples and representatives of local communities, and traditionally generated knowledge, must be included. These systems of knowledge can then be braided together with mainstream science to comprise a holistic framework for decision-making. Scholarship and methodologies being applied in other fields and contexts to solve complex challenges and manage uncertainty, such as collaborative governance, social-ecologic systems theory, and complexity science, must be recognized and incorporated. The spectrum of considered worldviews must also expand to authentically integrate the expanded scope and approach into action and sustainable impact. By increasing community and social engagement and by recognizing and entwining different worldviews, the plurality of disciplines, and traditional and scientific ways of knowing to address community concerns in the contexts in which they exist, we can ensure that One Health remains effective and true to its paradigm in our rapidly changing and complex world.

## 1. Introduction: One Health to today

Holistic, nature-based and society-based approaches to sustainably balance and steward human, animal, plant, and ecosystem health and well-being have been recognized and practiced since time immemorial by Indigenous communities, and continue to be lifeways for Indigenous Peoples (1–4). Other local and cohesive communities around the globe, such as self-proclaimed peasant, forest, and agrarian communities, often share similar, integrated worldviews (2, 4, 5). The value of such interconnected approaches to understanding the world has also been recognized from within mainstream scientific communities (6–8) and by national and international policymakers who must simultaneously address health, social, financial, and ecological crises (9–14).

In 2004, the Wildlife Conservation Society (WCS) identified the environment, including wildlife and ecological systems, as underrecognized yet critical components of complex emerging health issues in humans and animals (15). Sparked by global health crises in the late 1990s and early 2000s, the concept of addressing health issues at the human-animal-environment interface was widely promoted by the international agencies responsible for health (e.g., World Health Organization/WHO, World Organization for Animal Health/WOAH,^1^ Food and Agriculture Organization/FAO, United Nations System Influenza Coordination/UNSIC, United Nations Environment Program/UNEP, Convention on Biological Diversity/CBD), as well as by government and academic stakeholders in countries and regions across the world (16–19). Through wider application, the concept developed into what is now called “One Health,” which is currently widely recognized as a useful approach to addressing complex health issues (20–22).

A key tenet of the One Health approach has been inclusion of all relevant stakeholders,^2^ from conceptualizing and planning through implementation and assessment of the One Health activity. Broadly, One Health activities can be defined as any research, policy development and implementation, capacity and infrastructure development, and system building or strengthening activities relevant to the health and well-being of humans, animals, plants, or ecosystems. The assumption has been that multisectorality—having everyone at the table (with the further assumption that all have equal and equitable power, voice, and agency)—would improve the result and preclude making policy or technical decisions with unbalanced impacts, because the discussion among stakeholders would lead to compromise and alignment that would eventually balance all perspectives and needs equitably.

The One Health approach was adopted by many scientists, institutions, and organizations while still relatively nascent and theoretical. Through widening use and the availability of experience and best practices, the One Health approach evolved, matured, and became more robust. However, as a legacy of the zoonotic disease control objectives that inspired its widespread adoption and the backgrounds of the actors initially involved, One Health remains primarily applied to research, policy, and capacity development activities focused on addressing the emergence and spread of zoonotic diseases and antimicrobial resistance (AMR), and few activities labeled One Health have ventured beyond that scope in a meaningful way. The concepts of EcoHealth and Planetary Health have emerged and help to address the loss of the ecologic focus initially included by the WCS (23–25). Despite perceived variations in scope among these three concepts, One Health, at its foundation, is best considered to be any multisectoral, interdisciplinary approach aiming to strengthen trust, collaboration, communication, and coordination around a given complex health activity, and as such, applies equally well to activities conducted under both the EcoHealth and Planetary Health conceptual frameworks.

The One Health approach has been used in research, education, and policymaking to address complex health concerns at different levels and with different scopes, with slow yet steady progress in working more collaboratively across health sectors. Tools and guidance are available to support One Health activities in countries (26–28), and many countries have national One Health mechanisms responsible for health policy, strategy, and action (29–31). Recently, the United States Centers for Disease Control and Prevention and the Food and Agriculture Organization of the United Nations proposed an overarching One Health Operational Framework with integrated tools (32). Ecological and biodiversity considerations and Indigenous Peoples' traditional^3^ ways of knowing are increasingly influencing some One Health conversations (33–38). Importantly, Indigenous voices are also calling for more authentic inclusion in One Health (1, 3, 39–41).

This call to action introduces and proposes an important evolution and expansion of the current One Health approach across scope, approach, and worldview, to more effectively meet the challenges of our evolving and ever more complex world. It is meant to inspire those who are planning, developing, and implementing One Health activities, including the current One Health community and new partners, from Indigenous peoples and local communities to international policymakers. Specifically, we encourage broader engagement with and support for voices and knowledge from diverse contexts and other sectoral or disciplinary compartments to ensure that One Health remains robust, relevant, and true to its paradigm.

## 2. Evolving contexts, systems complexity, and their impacts on development and One Health

### 2.1. The evolving world context

Today's global crises can now only be ignored at great peril. There will be more pandemics, severe weather, irreversible ecosystem destruction, food insecurity, and widening health and well-being, social, and economic disparities for much of the world's population. These crises directly affect nations, communities, and individuals, and are rapidly reaching their tipping points. In parallel, there is an emerging recognition of the need for collective and equitable action toward a more ecologically sustainable and just future. To address the immense challenges of ensuring justice and equity while ensuring health and well-being, it is essential to recognize how limited worldviews and persisting systems of colonialism, discrimination, and oppression perpetuate and widen disparities, sustain community and environmental degradation, drive ecosystem collapse, and harm communities. These limitations also seriously threaten our ability to respond collectively to complex crises with sustained action. This situation has been well described for decades from myriad perspectives in published books (5, 35, 42–46) and scientific articles (36, 47–49). Moreover, diverse scientific and policy groups have repeatedly issued calls to action and statements proposing ways forward (35, 50–53).

From an economic and business perspective, the world is also evolving. Models of business maturity suggest that after a period of growth, businesses must evolve to account for current contexts, or else the normal trajectory is to become stagnant or obsolete. Bennett and Lemoine (54), among others, propose that the world is increasingly “Volatile, Uncertain, Complex, and Ambiguous” (VUCA) and subject to constant, unpredictable changes. The VUCA concept calls for more agile and responsive approaches to business and social processes, which could be equally well applied to health and ecosystem processes. In the context of the fourth industrial revolution as proposed by Klaus Schwab (55), new technologies will drastically change health care for individuals and communities (56). All these changes force continual and rapid reconsideration of current practices and opportunities across a range of social and health development activities, including One Health activities.

### 2.2. Complexity, sustainability, and negative externalities

Simultaneously, there is increasing recognition of connectivity and complexity in a wide variety of biologic and non-biologic systems. Meadows (57) defines complex systems as an interconnected set of elements that is coherently organized in a way that achieves a function or purpose. Systems complexity concepts have been well described, are being applied both inside and outside the health sector, and are clearly applicable and useful more broadly. As such, current crises are increasingly being examined with a complex systems perspective rather than a classic linear approach (57–60). Further, both academically and traditionally-generated scientific evidence is mounting to support that what we thought were complicated but generally straightforward biological ecosystems, such as forests, are far more interconnected and complex than imagined, with communication and interdependencies among organisms of not only different species, but also different kingdoms (61). Rovelli (62) proposes that “the world that we observe is continuously interacting. It is a dense web of interactions.” The result is that disruptions to the visible system can have unexpected and far-reaching impacts (58, 61).

Sustainability in the context of development has been defined as “meeting the needs of the present without compromising the ability of future generations to meet their own needs” (63). In this frame, positive outcomes of health and development activities within complex systems—including One Health activities—cannot be sustainable if, for example, only economic or health benefits are considered without simultaneously considering ecosystems, social systems, and cultures. However, health and development activities tend to be planned, funded, implemented, and assessed within a specific development topic or sectoral compartment (often referred to as a “silo”) to meet narrow objectives and highly specific targets or deliverables which do not consider the entire complex system and its evolving context. Such activities necessarily result in “negative externalities,” the term used commonly in economics for negative impacts that were not intended or fall outside the stated scope of the activity (and as such are only “external” relative to the intention). Negative externalities may be unpredictable, impossible to quantify, or both, but often affect the sustainability of activities and outcomes. Some may be relatively limited, like the increased plastic waste generated while providing broad local access to diagnostic tests. Others may be vast and not only affect the sustainability of the activity and its ability to meet its objectives, but even highlight or exacerbate inequities (64). For example, decarbonization projects such as wind farms may disrupt lives and livelihoods for Indigenous peoples and local communities, making sustainable and traditional activities and lifeways difficult or even impossible to practice (65). Such an emphasis on economic growth and technological progress, coupled with the structural devaluation of Indigenous and local expertise and cultural needs, may result in a net decrease in the health and resilience of the social-ecological system. In the example of the diagnostic testing program that generates plastic waste as a negative externality, a change in national plastic use policy may then make the program's continuation impossible even if it were otherwise successful. Notably, the relative size and scope of such negative impacts vary greatly depending on one's worldview (66).

It is likely that health and development activities that do not account for system complexity will experience negative externalities at some point (59). This may be especially true for activities with larger scopes and levels of complexity. More granular activities with limited interlinkages to the broader system may more easily identify all relevant stakeholders, proactively identify the range of impacts, and respond rapidly to unpredicted changes to mitigate negative externalities and thus may be better able to sustainably meet their objectives (67).

Unsustainability may also be related to a lack of resilience in the system (58). “Resilience” is defined as the capacity of a system, be it an individual, a forest, a city, or an economy, to absorb or adapt to disturbance and still retain its basic function and structure (58, 68). However, it can be challenging to determine what the basic function and structure of the system are or should be, what is considered a success or gain, and what the present and future needs are, as well as who gets to participate in making the decision.

### 2.3. Complex systems and the SDGs

The Sustainable Development Goals (SDGs) (69) are another example of how insufficient recognition of system complexity limits our ability to meet programmatic objectives. Most countries have embraced the SDGs, with some gains made, especially for highly specific targets (70, 71). Despite global enthusiasm and commitment and with recognition of the ambitious nature of the SDGs and setbacks due to COVID-19, tangible progress and global impact across the SDGs by 2030 seems unlikely. This is in part because, despite the importance of interlinkages and partnerships as recognized within the 2030 SDG Agenda, many current SDG activities focus on achieving a limited number of indicators within one or a few SDGs (72) rather than promoting and facilitating change across complex, vastly interlinked systems.

Further, development objectives may conflict. It has long been understood that progress in one development target may cause regression in others (73–75). For example, progress toward economic growth targets in SDG 8 might not be possible without negatively impacting other SDGs; concurrently considering and mitigating these impacts might then make it impossible to meet the economic growth targets (76). This situation is seen broadly in other economic development activities, especially as the current underlying model of development is one of consumption and infinite economic growth, which itself is inherently unsustainable (47, 77, 78).

The ubiquity of negative externalities and the dilemma of conflicting targets as seen in health and development contexts and the SDGs reflect the narrow, short-term focus with which development has typically been undertaken. Addressing these concerns and improving outcomes will require proactive changes in economic and development paradigms. These include a shift away from compartmentalized government, academic, scientific, and development infrastructure and policies to more long-term and equitable funding structures that recognize the complexity and unpredictability of the systems they are intended to impact. The possibility of new economic and business models brought about by these considerations would positively impact development models generally as well as improving implementation of the current One Health approach.

## 3. An evolution and expansion of the current One Health approach

### 3.1. Limitations of the current One Health approach

In planning and implementing One Health activities, insufficient recognition of system complexity and evolving contexts may affect the ability for objectives to be met. As a given health situation changes, even if it improves, specific actions and processes set up to address the original concern may no longer be relevant to the new circumstances and they may even undermine the evolving system unless it is resilient enough to adapt (58, 59). For example, a successful national One Health program for rabies control in stray dogs may be unsustainable if, as the burden of rabies in a location becomes less critical, the program is then defunded or set aside in the context of the larger national system of competing priorities, power shifts, and financial challenges (79). Short and medium-term gains are clearly important, but must be viewed within the larger ecological, social, and policy system from the beginning to ensure that they are balanced with sustainable, positive outcomes in the longer term.

Although a hallmark of One Health is inclusion of multiple partners, existing power imbalances within complex networks of stakeholders are often not considered or managed, resulting in inequitable outcomes. These imbalances have been shaped by histories and ongoing effects of colonialism and oppression and/or are based on the relative importance assigned to human, animal, plant, and ecosystem health. Wealth inequities and financial interests can also influence One Health activity planning and implementation, potentially undermining both equity and effectiveness. These imbalances and their resulting negative externalities impact the function and sustainability of One Health activities and outcomes.

We propose that these gaps and challenges result from inherent limitations in how the One Health approach is currently considered and applied across three interrelated dimensions. The first limitation is in *scope*, with overrepresentation from the health and biological sciences. Insufficient consideration is given to the other sciences that promote and sustain health and well-being, the political and social sciences and humanities, and the lived experience and traditional knowledge of communities and their cultural, economic, ecologic, and social contexts and realities (80). The second limitation is in *approach*, which is currently overwhelmingly linear, deterministic, and focused on the individual components of systems. Insufficient consideration is given to the uncertainty and connectivity that other fields—such as systems science and complexity science—already recognize and apply. The third limitation, in *worldview*, encompasses both scope and approach. Most activities labeled as One Health (e.g., zoonotic disease surveillance, epidemiological investigation, outbreak response) have been conducted within a mainstream scientific worldview (sometimes referred to as a “Western” worldview, despite also being dominant in Eastern parts of the globe). This worldview tends to be reductionist, with limited consideration of cultural norms and values, ethics, and personal or community belief systems and generally lacks respect for traditional knowledge generation methodologies, practices, or relational accountability (81).

### 3.2. An evolution of the current One Health paradigm

Given these limitations, we must evolve our One Health paradigm to remain relevant and effective in a world that is increasingly understood as a complex connection of systems. This evolution has begun. Calls to broaden One Health have already been made from inside and outside the One Health community (36, 82, 83) and systems and complexity concepts are increasingly mentioned within One Health publications (23, 84–86). The environment and ecology sectors are now being more functionally engaged in One Health at the international level (22, 87). The need for a new paradigm in international development is already well-known among many people working in complex contexts, especially within communities, and an evolved One Health paradigm could fulfill this need.

But there is more progress to be made. To address the limitations described above, we now propose a blending and braiding of knowledge and approaches to evolve and expand the One Health approach into a next iteration, a One Health Version 2.0. Braiding is a concept used by some Indigenous writers to emphasize the wholeness and separateness of different things that are nonetheless interconnected into another whole (46, 88). We are not proposing to forego the use of a mainstream scientific approach or to minimize the importance of using academically generated knowledge in our One Health activities. From here, we propose a need to assess and expand the One Health approach, particularly by increasing community (sometimes called “centering” community) and social engagement and by recognizing and entwining different worldviews, the plurality of disciplines, and traditional and academic ways of knowing to address community concerns in the contexts in which they occur.

This next iteration would maintain the best practices from current One Health principles and application while expanding in the following three dimensions.

#### 3.2.1. An evolution in scope

Currently, all descriptions of the One Health approach include the presumption of inclusivity and refer to including multiple stakeholders and the perspectives and knowledge they bring. However, in practice, this inclusivity tends to refer only to partners within the dominant or mainstream scientific contexts directly related to health. The One Health scope must broaden substantially to include a “system of systems” and all the associated partners including Indigenous peoples and local communities and other disciplines (e.g., biologic, social, political, economic). If we assume that the often-cited One Health Venn diagram, with the three overlapping circles representing humans, animals, and the environment, describes the scope of the health system, we can now envisage this system overlayed on myriad other interconnected systems, including climate/biodiversity/ecosystems, education/poverty/gender, social well-being/community/culture, economics/industry/development, etc. A diagram including some of these elements has recently been proposed by the One Health High Level Expert Panel (22), although it insufficiently emphasizes the interlinkages and interactions among the systems. Processes such as social network analysis (89) and inclusionary concepts such as “soft infrastructure”—where each aspect within the community system (including hope, trust, relationships, and worldview)—is holistically considered (90) could be useful in identifying and including all stakeholders and actors, including rights and title holders, within the system.

Once identified, existing power imbalances among all the actors must be acknowledged and managed. This will require a continued and intentional focus on justice and equity to ensure that: (1) each voice, especially those of marginalized communities and others without institutional power or agency is heard and valued; (2) that principles of free, prior, and informed consent (FPIC) for impacted communities are applied (91); and (3) that the ethical and value frameworks of the communities are not devalued but are understood, prioritized, and respected, even where they diverge from or contradict what is considered best practice by mainstream institutions (92). The stakeholders and actors that can bring these local considerations to the discussion will vary considerably across contexts so must be proactively identified from within the communities. Furthermore, One Health would benefit from further discussion and consideration of the pluralism of ethical principles and frameworks, including the ethical implications of unbalanced impacts and negative externalities on current and future generations resulting from the application—or lack of application—of a One Health approach (93–95).

The inherent nature of society and knowledge to continually change and evolve in profound ways over time also affects the scope of both the partners and the knowledge to include. There is increasing recognition that the marginalization of people based on ethnicity, gender, sexual orientation, and other identities^4^ has major impacts on people's lives and health. To address this, marginalized communities and their perspectives must be represented with proactive and sustained action. Indigenous knowledge and land stewardship, for example, has long provided foundational understanding of the complexities of local ecosystems, and Indigenous leadership and local organizations are at the forefront of conservation, interconnected pedagogy and ontology, and sustainable development (97). New revelations in diverse scientific realms about, for example, quantum effects in biological systems (98) and the role of fungi in sustaining global ecosystems (99) have the potential to change how we understand these systems. It is critical that we develop shared processes to equitably, efficiently, and consistently interweave these new partners, knowledge, and knowledge systems together with current and mainstream knowledge and knowledge systems into the One Health scope of consideration.

As the range and diversity of partners increases, understanding how to better encourage, govern, and maintain collaboration becomes increasingly important. Difficulties in working across sectors and disciplines are myriad, and compartmentalization spans the research cycle from funding to publication (100). Much scholarship and experience in both the theory and practice of taking multisectoral, transdisciplinary, or convergence approaches already exist, for example in the field of collaboration theory and through traditional knowledge generation and co-production of knowledge (80, 101–104). Notably, this important work on transdisciplinary collaboration is happening for the most part outside of current One Health activities, and those involved are not necessarily focused on “health” as an outcome (1, 40). The scholarship is generally neither considered nor referenced in One Health-specific publications, and some may not be published at all. Finding ways to identify, access, consider, and respect this information would add value and improve outcomes of the One Health approach.

#### 3.2.2. An evolution in approach

Given that One Health is applied to complex systems, simply expanding the scope of partners and knowledge (e.g., just including communities and local knowledge) would be insufficient without fundamentally changing the approach (e.g., considering these communities within their larger system). Complex systems, including biological and social-ecological systems, have unpredictable, non-linear properties such as feedback loops (where a change reinforces or balances further change), adaptation (adjustments made in response to interventions or information), and self-organization (the capacity of a system to make its own structure more complex) (57, 58, 60, 105, 106). Other properties of complex systems that add unpredictability include tipping points (where the outcome of interest appears resistant to efforts to improve it, until some critical combination of factors comes together and a transformative change occurs) and emergence (where a wholly unexpected outcome is expressed due to the interaction of different elements) (57, 58). These properties are further influenced by the resilience of the system (58).

Therefore, addressing components of complex systems with a linear approach is likely to be less effective than addressing or reshaping the complexity of interconnecting elements that define the system, especially as the system gets bigger and when contexts and impacts are considered over time. When planning for pandemics, for example, the research, policies, and practices related to physical, mental, and social health and well-being [which comprise the WHO definition of health, namely “… a state of complete physical, mental and social well-being and not merely the absence of disease or infirmity” (107)], as well as economics and politics, have historically been formulated within separate disciplinary systems and been based on the situation and context at that moment. Even mental and social well-being is inconsistently considered as an outcome in health planning, despite its contribution to the capacity to flourish in life. As a result of this limited, disjointed approach, pandemic planning fails to recognize and manage the complex and unpredictable interconnection and interplay of these dimensions of health and well-being, including impacts on mental health and social systems. For example, when standard public health risk mitigation measures were implemented during the COVID-19 pandemic, most communities were not prepared for the increases in mental health issues and domestic violence, which occurred due to increases in fear, isolation, and uncertainty. Further, while efforts were made to include community concerns and perceptions through community engagement in pandemic planning, political realities and the potential reactions of local and national policymakers in the evolving political environment were not considered as part of the system, resulting in the devastating nationalistic outcomes (e.g., prioritizing political over public health objectives) that ultimately undermined the public health measures implemented in many countries (108, 109).

One Health approaches must therefore move past mainstream scientific linearity and the current focus on a system's individual components. They must instead focus on a system's dynamic processes and interconnections and its functions and purposes, using leverage points to perturb the pattern of connections to influence the system, and adjusting when the system or context changes, irrespective of the constellation of components within the system. From a systems science perspective, first steps in the pandemic planning example would be to reassess the processes for planning and response decision making and identify leverage points to achieve the desired impacts. These considerations would be based on the overall purposes of the system (be it optimal health and well-being of the human population or something else), and the processes would be balanced to account for the power dynamics within the system. Inclusion of policymakers as integral parts of the system would facilitate the development and implementation of joint decisions and the forecasting of changing policy. Inclusion of a wider scope of actors and stakeholders' groups would allow target outcomes to include the optimal health and well-being of humans, animals, plants, and ecosystems. In all instances, rather than describing what should be done and by whom for each relevant objective, the systems-focused plan would describe the processes for evaluating the system, deciding what would be done and who to involve, determining if the objectives were being achieved, and making decisions to reevaluate and redirect as needed. First steps in planning for the wind farm example, above, would establish processes to proactively consider the interconnections between the ecologic system; the impacts to Indigenous culture, knowledge, norms and values; the local and national policies; the power imbalances; and the social landscape. Planners would consider how and why these interconnections might change and how they have historically been impacted and would establish how FPIC by the impacted community would be ensured, in order to maintain the social-ecological system as a coherent whole. In both examples, finding ways to improve or preserve the resilience of the system would make it less prone to perturbations overall, with the goal shifting from making the activities sustainable to making the framework of processes sustainable as well as resilient.

Resilience, adaptive and emergent properties, interconnections, and complexity of systems have been well described in the intersecting fields of systems science, social-ecological systems analysis, and complexity science (57, 58, 106), and these concepts have already been applied to social, biological and ecological systems (57, 60, 110–116). Even quantum mechanics is proving useful to resolve certain longstanding quandaries in biological processes, such as the efficiency of photosynthesis and geolocation in migrating birds (98), and it makes sense that quantum processes happening at sub-atomic particle level in biological systems would lay the foundational groundwork for how larger biological processes work. Chaos theory proposes that tiny differences in starting values can lead to drastically different outcomes and can help us think about how iterations of simple—even apparently deterministic—processes can generate unpredictability (98, 101).

With some recognition of these concepts, it becomes easier for scientists trained within mainstream academic science to accept the enormous complexity and unpredictability of our world and resist the urge to try to simplify it. These concepts (i.e., complex systems science, quantum mechanics, chaos theory) can help us to understand that complex systems—biologic and non-biologic alike—are primarily non-linear and change in unpredictable ways. They allow us to view this unpredictability using a probabilistic rather than (or in addition to) a deterministic lens, to better plan for and find ways to accept and work within uncertainty and to manage unpredicted changes with more flexibility and resilience. We can notice that small changes in one system can have enormous impact elsewhere, even if the exact cause and effect cannot be predicted or elucidated. As each of these areas of work already provides scholarship and tools for thinking methodologically, we can already start to explore their application in One Health contexts in a methodologically rigorous way.

As the potential applications of these evolving concepts to One Health are new and may be completely unfamiliar to many, it is critically important to be diligent about terminology. Definitions and practical use of terms such as systems thinking, complexity theory, quantum biology, complexity science, and chaos theory vary widely, as does the terminology used within these rapidly growing areas of work (106, 111, 117). Parrish-Sprowl et al. (115) note that in some instances, the complexity of the problem may be recognized and stated, yet the concepts and theory behind the activities may remain linear and reductionist. This contradiction can undermine interpretation of the application of the One Health approach and its value, as the system's complexity is not actually being addressed. Ensuring terminology is clearly defined in context will be critical to effectively conduct and share research and test the application of these concepts within an expanded One Health approach.

#### 3.2.3. An evolution in worldview inclusivity

Worldviews frame how knowledge and expertise are created, used, cared for, and sustained. The inherent worldview assumptions that are attached to any activity shape the questions, purpose, activities, and eventual outcomes of the activity. The mainstream scientific worldview which currently predominates in health and development—including One Health—was founded on Newtonian and Cartesian epistemologies and assumes a cause-and-effect linearity without explicitly considering the diversity of values, beliefs, and ethical frameworks, or providing for any uncertainty or complexity that cannot be predicted or adjusted for through rationalism. Mainstream science also divides knowledge into separate fields which not only perpetuates compartmentalization of worldviews and further constrains holistic thinking, but it also requires new learners to gain expertise in a compartmentalized way *via* educational institutions and credential programs (118).

Alternate systems of knowledge production are organized around different constructions of knowledge and expertise based on culturally relevant factors. Indigenous knowledge systems have been developed over tens of thousands of years and include systems for building learners' expertise and responsibility to the social and ecological systems around them (119). Indigenous science cannot be singularly defined because each Indigenous community has a unique system that prioritizes their relevant lifeways, cultures, and languages (120, 121). However, although these knowledge systems were created with different core cultural values, they each include empirical data gathering, experimentation, preferred pedagogical methods, and a system to create expertise and leadership in decision making (118, 119). Such knowledge systems are not simply data-driven but are themselves detailed social and ecological systems carefully maintained to support communities and their surrounding ecosystems. Similarly, cohesive communities that are local to a particular place, but not necessarily Indigenous, often have such systems of experimentation and empirical observation consistent with their lived experiences. Community rules, values, spirituality, and customs are considered in these systems, as these will impact the success and sustainability of any new activity and its outcomes. Intentional or unintentional actions that undermine community stewardship or sovereignty can further injustices and potentially destroy ecosystems and community cohesion.

Despite external influences and the long history and ongoing violence of colonialism, extraction, and racialized violence against Indigenous peoples, their worldviews, and the practices that emerge from their knowledge systems, Indigenous and other marginalized communities continue to perpetuate their knowledge systems and worldviews. They do this both within their communities and as thought leaders in addressing global issues like climate change, health, food sovereignty, and collaborative governance (122–124). Further, Indigenous leaders throughout the globe are reimagining and revitalizing their cultures and languages, advocating for broader implementation of Indigenous knowledge, and asserting sovereignty and self-determination over their futures (125). Other marginalized communities have similarly created and maintained community through shared decision-making and collective action toward justice. For example, some communities of color in the USA have created and continue to lead movements for environmental justice that prioritize health, community voice, and ecosystem health (126–128).

These movements and their leaders have much experience, knowledge, and perspectives to offer One Health activities through a justice-oriented and holistic approach. However, to effectively include community and Indigenous leadership in a real way, One Health activities must expand to bridge multiple worldviews. The first step to authentically engaging Indigenous worldviews, for example, would be to eliminate barriers for effective Indigenous leadership and governance and to partner in true collaboration with communities using co-design and co-conception from an activity's inception (129, 130). A partnership that includes multiple worldviews will need to acknowledge the inherent power dynamics between mainstream, academically generated science and community-driven knowledge systems. Holistically including additional worldviews will require a framework such as “Two-Eyed Seeing,” championed by Mi'kmaw Elder Dr. Albert Marshal and his wife, which approaches Indigenous knowledge and mainstream science as equals and suggests that collaborations should attempt to see through both “eyes” instead of through one or the other to comprise an expert and holistic system of knowing and understanding the world (131, 132). Both traditional and mainstream approaches (and others that may emerge as new partners are engaged in specific activities) are essential as One Health broadens its collaborations and seeks to engage diverse worldviews more fully (133). Simply recognizing that different worldviews exist may facilitate conversations among partners by allowing time and space for expression, explanation, shared understanding, co-learning, un-learning, and forward-thinking solutions (134).

These proposed expansions of scope, approach, and worldview and the blending and braiding of diverse approaches together would allow a given activity to be more authentically considered and addressed, so that planning and decision-making could be contextualized wholly within the appropriate community or system, to ensure that One Health activities continue to sustainably shape a more healthful and just future (103, 135).

## 4. Moving forward

We have highlighted the changing world and current global crises and recognize that the imminent degradation of the quality and diversity of life on earth now requires a strong, sustained, and harmonized engagement of partners across levels, working together using innovative approaches and with respect for and consideration of multiple worldviews. We see value in authentically including Indigenous peoples and local communities, recognizing the inherent properties of complex systems, promoting both health and well-being of human, animal, plants, and ecosystems as outcomes, recognizing and braiding together the multiplicity of worldviews, and approaching science through both traditional and academic ways of knowing in order to address community concerns in the contexts in which they exist.

Specifically, we propose a broadening of scope, an expansion of approach, and the inclusion of diverse worldviews in a next iteration of One Health to facilitate addressing current constraints to resilience and the sustainability of positive outcomes in health and development. We humbly recognize that at this point, this author team cannot propose a specific way forward in any of these directions, in large part because specific challenges require tailored solutions to be explored and co-created with the appropriate scope of partners. Part of this call is to those methodologists (e.g., in complex adaptive systems science, in governance theory) and to Indigenous experts and traditional knowledge keepers to help us carry the conversation forward.

The process for expanding One Health's scope, approach, and worldview could be a model for the new approach itself. Proactive efforts to understand the complex systems we work in and applying a lens of inclusivity will provide opportunities for improved communication and allow us to access knowledge, experience, and practice currently compartmentalized within sectoral or disciplinary silos that we may not yet have identified. We see opportunities to newly apply and test these practices in One Health settings and to further research and operationally explore effective ways of bringing people and knowledge together. We recognize worldview-related limitations in valuing knowledge and knowledge systems, and here propose that ongoing development and incorporation of new knowledge means not just knowledge generated through mainstream academic processes, but also through traditional and non-linear approaches, proactively including perspectives from community voices and the plurality of sectors and disciplines.

There are benefits to having standard definitions for One Health and related approaches, but only when terms are used inclusively. We propose that any terminology applied to the One Health evolution (or “Version 2.0”) described here would consider the physical, mental, and spiritual health and well-being of all living human and non-human beings and ecosystems as interlinked across the globe, and would be translatable across languages and cultures.

We encourage and invite anyone who finds the ideas proposed here useful to contribute to the proposed evolution and expansion of One Health. The engagement of organizations and institutions that currently work primarily in sectoral or disciplinary compartments or silos and contribute to compartmentalized infrastructure at any administrative level would help to promote true and sustained change. We hope to build a coalition of individuals, communities, and institutions with diverging perspectives and worldviews who believe that we can and must rapidly and fundamentally change our approach to health, development, and ecology. We see enormous opportunities for further exploration of the principles presented here. Much thinking and options for practical implementation are left to be further developed, discussed, and implemented together with communities throughout the world.

## Author's note

The following authors are members of the One Health Action Collaborative at the National Academies of Sciences, Engineering, and Medicine: GH, JM, EliM, DR, JP-S, and ET.

## Author contributions

EliM, ElaM, and JP-S led the conceptualization of the manuscript. EliM formulated and drafted the manuscript except the section on worldview which was prepared by DM with concept and literature support by KT-H. All authors contributed conceptually or with specific content or editing and all approved the final manuscript.

## Footnotes

^1^Previous acronym was “OIE” https://www.woah.org/en/the-world-organisation-for-animal-health-launches-its-refreshed-brand-identity/.

^2^As defined in WHO (22).

^3^In this paper “traditional” is defined as historical and/or contemporary knowledge, innovations, and practices of Indigenous peoples and local communities around the world.

^4^Respecting continued concerns with using these terms (96).
